# Spina Bifida

**Published:** 1891-01-10

**Authors:** 


### SPINA BIFIDA.

These cases were formerly looked on as practically incurable, puncture, before the introduction of antiseptic treatment, having given very unfavourable results, and the merely palliative employment of support having been preferred. But surgeons have latterly undertaken them with greater success. Dr. Julius Lindemann in his inaugural dissertation, describes one of the lower lumbar vertebræ, the size of a hen's egg, in a child aged 17 months. It was cut off across the base and the membranes of the sac sutured. Erysipelas followed, but the recovery was complete. This operation is, we think, not to be recommended, for had nerve trunks been adherent to the membranes, the consequences might have been serious. A more cautious procedure was followed by Dr. Borelius in the case of a large lumbo sacral meningocele in a child aged 10 months. Two lateral flaps of skin having been made, the sac was punctured, and the fluid drawn off. The walls were carefully examined, and no nerves being seen in them, the greater part of the sac was excised and the margins left were joined by suture. The skin flaps were in like manner reduced in size, brought together and sutured. Unfortunately the child died two months later of measles. but, except in two small points, the wound had completely healed.

